# Resilience Is Associated with Less Eating Disorder Symptoms in the NutriNet-Santé Cohort Study

**DOI:** 10.3390/ijerph19031471

**Published:** 2022-01-27

**Authors:** Margaux Robert, Rebecca Shankland, Valentina A. Andreeva, Mélanie Deschasaux-Tanguy, Emmanuelle Kesse-Guyot, Alice Bellicha, Christophe Leys, Serge Hercberg, Mathilde Touvier, Sandrine Péneau

**Affiliations:** 1 Université Sorbonne Paris Nord, Inserm U1153, Inrae U1125, Cnam, Equipe de Recherche en Epidémiologie Nutritionnelle (EREN), Centre de Recherche en Epidémiologie et Statistiques–Université de Paris (CRESS), 93017 Bobigny, France; v.andreeva@eren.smbh.univ-paris13.fr (V.A.A.); m.deschasaux@eren.smbh.univ-paris13.fr (M.D.-T.); e.kesse@eren.smbh.univ-paris13.fr (E.K.-G.); a.bellicha@eren.smbh.univ-paris13.fr (A.B.); s.hercberg@eren.smbh.univ-paris13.fr (S.H.); m.touvier@eren.smbh.univ-paris13.fr (M.T.); s.peneau@uren.smbh.univ-paris13.fr (S.P.); 2 Laboratoire DIPHE (Développement, Individu, Processus, Handicap, Education), Université Lumière Lyon 2, 69000 Lyon, France; rebecca.shankland@univ-lyon2.fr; 3 Service D’analyse des Donnees (SAD), Université Libre de Bruxelles, 1000 Bruxelles, Belgium; christophe.leys@ulb.be; 4 Département de Santé Publique, Avicenne Hospital, 97017 Bobigny, France

**Keywords:** resilience, eating disorders, positive psychology, epidemiology, longitudinal study

## Abstract

Resilience is a positive psychological trait associated with a lower risk of some physical and mental chronic diseases and could be an important protective factor against eating disorders (EDs). The aim of this study was to assess cross-sectional and longitudinal associations between resilience and ED in a large cohort of French adults. In 2017, a total of 25,000 adults from the NutriNet-Santé cohort completed the Brief Resilience Scale (BRS). ED symptoms were measured in 2017 and 2020, with the Sick-Control-One-Fat-Food (SCOFF) questionnaire. Cross-sectional and longitudinal associations between resilience and EDs were analyzed using logistic regression, controlling for sociodemographic and lifestyle characteristics. Cross-sectional analyses showed that more resilient participants exhibited EDs less frequently than did less resilient participants (*p* < 0.0001). Longitudinal analyses showed that, during the three years of follow up, higher resilience was negatively associated with incident EDs (OR: 0.67, 95%CI: 0.61–0.74), persistent EDs (0.46 (0.42–0.51)), and intermittent EDs (0.66 (0.62–0.71)), compared with no ED. More resilient participants were also less likely to have a persistent ED than to recover from EDs (0.73 (0.65–0.82)). This study showed that resilience was associated with less ED symptoms and a higher chance of recovery.

## 1. Introduction

Eating disorders (EDs) are defined as “persistent disturbances of eating or eating-related behaviors that result in the altered consumption or absorption of food and that significantly impair physical health or psychosocial functioning” [1]. A review of studies conducted in various countries indicated a lifetime prevalence of EDs of 8.4% for women and 2.2% for men [2]. EDs are generally of long duration: 5–8 years on average for bulimia and binge eating disorders, and around 2 years for anorexia nervosa [3,4], and have important consequences for physical and mental health, such as low bone mineral density [5], anxiety disorders [3], depression [6,7] and/or substance abuse [3]. Individuals with EDs also have a higher odds of premature death [8], partly due to an increased risk of attempted suicide [8,9]. It is therefore important to identify and understand the risk and protective factors of EDs, in order to guide prevention. While risk factors have been widely studied [10,11,12], research on protective factors is more limited. In particular, positive psychology, which aims to expand the focus of psychology from only addressing the negative aspects in life to building an individual’s positive assets [13], could be a pertinent resource in the prevention of EDs [14]. Building competencies instead of correcting weaknesses has been identified as an important contributor to the major strides in prevention [13]. Resilience, defined as the process of adapting well in the face of adversity, trauma, tragedy, threat or important sources of stress [15], is one of those positive psychological traits. It has been inversely associated with several physical and mental health outcomes, such as a decreased risk of developing type 2 diabetes [16], cardiovascular disease, [17,18] and cancer [19]; increased longevity [20]; and lower risk of anxiety [21] and depression [22]. We hypothesized that resilience might also play a role in the onset and course of EDs, due to its association with positive coping strategies when facing stressful situations [23,24] and with a reduced likelihood of body dissatisfaction [25,26]. Yet, only a few studies have investigated the associations between resilience and EDs. Cross-sectional studies found that resilience was lower in people diagnosed with EDs [27,28,29,30], more specifically with anorexia nervosa [27], bulimia nervosa [27] or binge eating [29]. However, to our knowledge, no study has yet explored the longitudinal association between resilience and incident EDs in a general population. Resilience has been identified as a criterion for ED recovery [31,32], but data are still scarce. In addition, since EDs are often considered to primarily affect adolescent and young adults, studies on ED have been largely conducted among these populations [33,34,35]. However, prevalence of ED among adults is not negligible [36,37,38], which justifies studying risks and protective factors of ED in an adult population.

The objective of this study was therefore to assess the cross-sectional and longitudinal associations between resilience and EDs in a large cohort of French adults, accounting for sociodemographic and lifestyle characteristics.

## 2. Materials and Methods

### 2.1. Study Population and Design

This study was conducted as part of the French NutriNet-Santé cohort study. This ongoing web-based cohort was launched in 2009 with the aim to examine the associations between nutrition and health, as well as the determinants of nutrition-related behaviors. The rationale, design and methods have been described elsewhere [39]. Participants are French adult volunteers (aged 18 and older). They complete web-based questionnaires to assess their diet, anthropometric status, lifestyles characteristics, socioeconomic conditions, physical activity and health status at inclusion and each year after inclusion. In addition, complementary questionnaires related to determinants of eating behaviors, nutritional status and specific health-related aspects are sent to participants each month.

The NutriNet-Santé study is conducted in accordance with the Declaration of Helsinki, and all procedures were approved by the Institutional Review Board of the French Institute for Health and Medical Research (IRB Inserm n◦ 0000388FWA00005831) and the Commission Nationale de l’Informatique et des Libertés (CNIL n◦ 908450 and n◦ 909216). Electronic informed consent was obtained from all participants. The study is registered at clinicaltrials.gov as #NCT03335644.

### 2.2. Assessment of Resilience

Resilience was measured between January and July 2017 with the French version of the validated Brief Resilience Scale (BRS) screening tool [40]. The BRS is a self-report questionnaire composed of 6 items: 3 positively worded statements (e.g., “It does not take me long to recover from a stressful event”) and 3 negatively worded statements (e.g., “I tend to take a long time to get over set-backs in my life”). Each item is scored on a 5-point Likert scale, ranging from 1 (“strongly disagree”) to 5 (“strongly agree”). The scoring for the negatively worded items was reversed and added to the score of the other items. The resulting score was divided by the total number of items, leading to a final score ranging from 1 (low resilience) to 5 (high resilience). In our study, the scale showed good internal consistency (Cronbach’s α = 0.84).

### 2.3. Assessment of Eating Disorder (ED) Symptoms

ED symptoms were assessed with the validated French version [41] of the Sick-Control-One-Fat-Food (SCOFF) questionnaire [42], administered once between April and October 2017, and a second time between March and September 2020. The SCOFF has good sensitivity and specificity regarding the detection of ED [41,42,43]. This self-report questionnaire includes five dichotomous items (e.g., “Do you worry you have lost control over how much you eat?”) (Yes = 1/No = 0). The scoring of the questionnaire assigns one point for every “yes” and a total score ≥ 2 (out of 5) indicates ED symptoms. To distinguish the different types of EDs, we used the Expali™ algorithm [44], which takes into account each SCOFF response and the individual’s body mass index (BMI) to categorize participants into four broad ED categories based on the DSM-5: (a) restrictive disorders category, including anorexia nervosa, restrictive food intake disorder and atypical anorexia nervosa; (b) bulimic disorders category, including bulimia nervosa or bulimia nervosa of low frequency or duration; (c) hyperphagic disorders category, including binge-eating disorders and binge-eating disorder of low frequency or duration; (d) other ED category, including purging disorder, night eating syndrome, and any other EDs.

BMI (kg/m^2^) was calculated as the ratio of self-reported weight (kg) to squared self-reported height (m^2^). Our anthropometric questionnaire has shown good validity [45,46]. We used the mean BMI of all weight/height values reported by participants during a time window comprising 2 years preceding and 6 months following the completion of each SCOFF. BMI was classified into six categories according to the WHO reference values [47]: underweight (BMI < 18.5 kg/m^2^), normal weight (18.5 ≤ BMI < 25.0 kg/m^2^), overweight (excluding obesity) (25.0 ≤ BMI < 30.0 kg/m^2^), obese class I (30.0 ≤ BMI < 35.0 kg/m^2^), obese class II (35.0 ≤ BMI < 40.0 kg/m^2^), obese class III (BMI ≥ 40.0 kg/m^2^).

### 2.4. Covariates

Data on potential confounders of the association between resilience and ED symptoms were collected each year. We used the latest data available prior to the completion of the BRS. Collected data included age (years), sex (men, women), educational level (primary, secondary, undergraduate, and postgraduate), occupational status (unemployed, student, self-employed and farmer, employee and manual worker, intermediate professions, managerial staff and intellectual professions, and retired), equivalized monthly household income, family situation (living alone without children, living alone with children, living in a couple without children, living in a couple with children), smoking status (current, former, and never smoker) and physical activity. Equivalized monthly household income was calculated using information about income and household composition. The number of people in the household was converted into a number of consumption units (CU) according to the OECD (Organization for Economic Cooperation and Development) equivalence scale: one CU is attributed for the first adult in the household, 0.5 for other persons aged 14 or older and 0.3 for children under 14 [48]. Categories of monthly household income were defined as follows: <1200; 1200–1799; 1800–2699; and ≥2700 euros per household unit as well as “unwilling to answer”. Physical activity was assessed with the short form of the French version of the International Physical Activity Questionnaire [49]. Weekly energy expenditure, expressed in Metabolic Equivalent of Task in minutes per week (MET in minutes/week), was estimated and three levels of physical activity were defined: low (<30 min/day), moderate (30–60 min/day), and high (≥60 min/day).

### 2.5. Statistical Analyses

Student *t* test and Chi-squared test were used to compare included and excluded participants. Relationships between resilience levels and baseline individual characteristics were analyzed with Pearson correlations for continuous variables and Student *t* test and variance analysis (ANOVA) for categorical variables.

The cross-sectional and longitudinal associations between resilience (independent variable) and EDs (dependent variable) were assessed with binary (yes vs. no) and multinomial (categories of EDs) logistic regression models. For the longitudinal analyses, we split our sample into four subgroups: “No ED” at either time point, “Incident ED” (2017: no ED, 2020: ED), “Recovered from ED” (2017: ED, 2020: no ED) and “Persistent ED” (2017: ED, 2020: ED). An additional “Intermittent ED” subgroup was built comprising both individuals of the “Recovered from ED” and “Incident ED” grouped together. First, we compared the “Incident ED”, “Persistent ED” and “Intermittent ED” groups with the “No ED” group. Second, we compared the “Persistent ED” group with the “Recovered from ED” (any ED) group.

Analyses were not stratified by sex because interactions with resilience were non-significant (all *p* > 0.20). All variables associated with resilience and EDs at the *p* < 0.20 level in the bivariate models were retained as confounders in the multivariable logistic regression analysis. The first model was unadjusted, and the second model was adjusted for age, sex, educational level, occupational status, equivalized monthly household income, family situation, smoking status and physical activity.

Sensitivity analyses were conducted after excluding participants who completed the SCOFF after 17 March 2020 (start of the COVID-19 lockdown in France).

All tests of statistical significance were 2-sided and significance was set at 5%. Missing data on confounders were handled with multiple imputations by a fully-conditional specification (20 imputed datasets). Statistical analyses were performed using SAS software (SAS Institute Inc., version 9.4, Cary, NC, USA).

## 3. Results

### 3.1. Characteristics of the Sample

A total of 37,620 participants of the NutriNet-Santé cohort completed the BRS, out of 118,707 participants who had received it. A total of 89 participants were excluded due to acquiescence bias (agreeing with all statements without consideration of reverse wording). Among those remaining, we excluded 3627 participants because they had not completed the SCOFF in 2017; 56 participants because they did not have valid anthropometric data close to the SCOFF administration in 2017; 8530 participants because they did not complete the SCOFF in 2020; and 318 participants who had one type of ED in 2017 and a different type of ED in 2020. Thus, we obtained a final study sample of 25,000 participants.

Compared with participants in the NutriNet-santé cohort who did not complete the BRS and were thus excluded from the present analysis, included participants were older (45.8 ± 14.1 years for excluded participants vs. 55.0 ± 14.5 years for included participants, *p* < 0.0001), included a higher proportion of men (20.9% vs. 25.7%, *p* < 0.0001), and of individuals with university education (61.2% vs. 63.2%, *p* < 0.0001). They were also more likely to have a high equivalized monthly household income (≥2700€) (18.8% vs. 33.6%, *p* < 0.0001), to live as a couple with children (50.6% vs. 63.2%, *p* < 0.0001), to have higher levels of physical activity (28.2% vs. 38.3%, *p* < 0.0001), and to have never smoked (46.7% vs. 50.6%, *p* < 0.0001).

Table 1 shows individual characteristics of the sample and their association with resilience. Overall, the mean score for resilience was 3.31 ± 0.69. Resilience was higher in men, in older individuals, in participants with a lower level of education, in those who were self-employed, farmers, managerial staff, had intellectual professions or were retired, in individuals with higher monthly income, living alone with children, former or current smokers, and in individuals with a higher level of physical activity (all *p* < 0.0001). In addition, the proportion of participants who had ED symptoms was 10.2% in 2017, and 8.8% in 2020.

### 3.2. Association between Resilience and EDs

Table 2 shows the results of the cross-sectional association between resilience and EDs in 2017. More resilient participants were less likely to have ED symptoms overall, and all types of EDs: restrictive, bulimic, hyperphagic, and other disorders. For example, for a one-point increase in resilience, the OR for ED symptoms was 0.53 (95%CI: 0.5, 0.56) (model 2). Overall, model 1 and 2 showed similar results.

Table 3 shows the results of the longitudinal associations between resilience and incident, persistent and intermittent EDs. After the three-year follow up, compared to the no-ED group, more resilient participants were less likely to have incident ED, especially incident bulimic, hyperphagic or other EDs. No association was observed for restrictive disorders. In addition, compared to the no-ED group, more resilient participants were less likely to have persistent ED, in particular restrictive, bulimic and hyperphagic disorders. No association was observed for other EDs. More resilient participants also were less likely to have an intermittent ED than no ED during the follow up. In particular, they were less likely to have all types of EDs: restrictive, bulimic, hyperphagic and other eating disorders. These results were similar in model 1, with the exception of the associations between resilience and restrictive incident and persistent disorders, which were significant.

Table 4 shows the results of the longitudinal associations between resilience and recovery from EDs. Overall, more resilient participants were less likely to have persistent EDs after three years than to have recovered from EDs, particularly for restrictive, bulimic and hyperphagic disorders. No association was observed for other types of EDs. Model 1 showed similar results.

### 3.3. Sensitivity Analyses

Further analyses were conducted, excluding participants who responded to the SCOFF 2020 after 17 March 2020, which was the beginning of the COVID-19 lockdown in France, and showed no substantial change of the results (N = 10,935). Only the association between resilience and the “persistent restrictive disorder” group, compared with the “recovered from restrictive disorder” group, became non-significant (*p* = 0.21).

## 4. Discussion

To our knowledge, the present study is the first to investigate the longitudinal associations between resilience and ED symptoms. Our results showed that higher resilience was significantly associated with less ED symptoms, both cross-sectionally and longitudinally. Particularly, compared to individuals without ED at either time point, individuals with higher resilience were less likely to have experienced an ED during the 3-year follow-up, either incident, persistent, or intermittent. In addition, they were less likely to have a persistent ED than to recover from an ED.

### 4.1. Level of Resilience According to Sociodemographic and Lifestyle Characteristics

The overall resilience score observed in our study is consistent with other studies in the literature [29,50,51]. Likewise, our findings are consistent with previous data indicating higher resilience levels in men [52], older individuals [52,53], and participants with higher income [54]. In our sample, however, resilience was higher in individuals with lower educational levels, in contrast with a previous report [52]. We also found that resilience levels were higher in self-employed individuals, farmers, managerial staff, in those with intellectual professions and in retired participants. Individuals with higher levels of physical activity and those living alone with children also displayed higher resilience scores.

### 4.2. Association between Resilience and Eating Disorders

In agreement with previous reports in the literature [27,28,29,30], our cross-sectional analyses showed that higher resilience was associated with lower ED symptoms. In particular, more resilient participants were less likely to have restrictive, bulimic or hyperphagic disorders, consistent with previous data reporting lower levels of resilience in patients with anorexia [27], bulimia [27] or binge eating disorder [29]. In addition, another study identified resilience as a mediator of the association between family types and the occurrence of EDs [55]. In that study, the so-called “balanced families” (more functional) were more resilient than “extreme families” (less functional), and more resilient families had less EDs. Our longitudinal analyses were consistent with our cross-sectional results and provided further support for the association between resilience and EDs during the 3-year follow-up. More resilient participants were less likely to have a persistent ED (restrictive, bulimic or hyperphagic disorders) and an intermittent ED (all categories) compared with no ED. In addition, more resilient participants were less likely to have incident ED at follow-up, in particular incident bulimic, hyperphagic or other disorders.

ED development is influenced by personality and mental states. For example, a combination of neuroticism and introversion have been suggested to be risk factors for symptoms of EDs in young women [56]. Meanwhile, resilience has been negatively associated with neuroticism [24] and introversion [24]. In addition, anxiety and depression can be precursors to the development of EDs [7,11], whereas resilience has been shown to be associated with less negative emotions [21,22], leading resilient individuals to be at potentially lower risk for anxiety and depression compared to their counterparts lacking this trait [57]. The experience of traumatic events during childhood is another risk factor for EDs. As resilience is associated with positive coping strategies [23], we suggest that resilient individuals might cope better with traumatic events occurring throughout life, and thus be at lower risk of developing EDs. The latter can also be caused by body dissatisfaction [11], with the thin ideal contributing to extreme weight control that characterizes anorexia nervosa and bulimia nervosa [12]. EDs have also been correlated with dieting [11], which has been shown to be negatively associated with resilience [25,26]. Peer influence is also a known risk factor for EDs [11,12,33]. For example, in women, having a college roommate who was dieting significantly predicted drive for thinness and bulimia incidence ten years later [58]. Friends’ or parents’ dieting has also been suggested as a potential predictor of body dissatisfaction [33], constant dieting [59], unhealthy/extreme weight control behavior [59] as well as binge eating in adolescents [59]. In addition, it has been suggested that individuals tend to associate with peers with similar personality [60,61]. By extension, individuals with EDs might associate with peers who are also vulnerable to EDs, which can influence their own ED status [60]. It is possible that resilient individuals would tend to socialize with other resilient individuals or with those with positive mental states, who are at lower risk for EDs and therefore have a positive influence on their eating behavior.

We also found that more resilient participants were more likely to recover from EDs than having a persistent ED after three years, especially in the case of restrictive, bulimic and hyperphagic disorders. These results are in accordance with previous longitudinal data indicating that resilience predicted a reduction of ED over time [32]. In addition, a qualitative meta-analysis suggested that resilience should be considered as a fundamental criterion of ED recovery [31]. As previously mentioned, resilience is associated with positive coping [23], which can be a great resource for recovery, as it may help individuals cope with the stress and/or trauma caused by a past or current event, that may reinforce their EDs. Besides, resilient people tend to have greater social support [62], which can help those suffering from ED to seek help and support them in their recovery journey. Resilience is also associated with a better quality of life [32], which is itself suggested to be a resource in the achievement of recovery [63].

Women are more affected by EDs than men [2], which could suggest differential mechanisms in the association between resilience and EDs, according to sex. Yet, interactions between resilience and sex were non-significant, suggesting a similar effect of resilience on EDs in men and women.

Various interventions are available to increase resilience, and can be delivered to groups or individually [64]. For example, the SMART program [65] designed to enhance resilience, focuses on two aspects: attention and interpretation. During the program, participants attend group sessions during which they are taught to focus their attention on the external world and to cultivate and guide their interpretation by five higher-order principles: gratitude, compassion, acceptance, meaning, and forgiveness. The SMART program has been shown to be effective in increasing resilience [65,66].

### 4.3. Strengths and Limitations

The main strengths of this study are its longitudinal design and its large sample, with individuals of various socio-demographic backgrounds which allowed the use of multiple covariates in the adjusted analysis. However, we cannot rule out the possibility that other important confounders were not considered in the present analyses. Our study could also present a selection bias due to the sampling strategy based on volunteering. Our sample comprised more women and more participants with higher education, higher income and professional status than the general French population [67]. In addition, participants are possibly more likely to have high health awareness and a stronger interest in nutrition. The selection bias indicates that caution should be exercised when extrapolating results to the general population. However, the mean resilience score observed in our study was consistent with scores observed in other studies [29,50,51]. This level of resilience was measured with the BRS, which has been validated [40] and demonstrated good psychometric properties in our study. ED symptoms were assessed with the SCOFF, which has been shown to be less effective in general populations than at risk populations, with a good specificity but a low sensitivity [68,69]. However, this tool has been previously recommended for screening purposes [43]. Since it has been demonstrated that the efficacy of the SCOFF increases as the percentage of women in the sample increases [43], the large proportion of women (74%) in our study is an advantage. The use of the Expali™ algorithm is another strength since it allowed us to distinguish the main categories of ED. A limitation of our study is linked to the temporal ordering of the data, as the second SCOFF was administered between during the COVID-19 pandemic which led to a lockdown in France from 17 March–5 May 2020 which may have resulted in psychological and behavioral changes [70]. However, the items of the SCOFF are worded in a way that suggests chronicity of the behavior, indicating that this questionnaire would screen for established behaviors and possibly underestimate recently emerged EDs. In addition, sensitivity analyses were conducted including only participants who completed the SCOFF before the lockdown onset and indicated similar results although the statistical power was lowered. Finally, to assess the significance of our results from a public health perspective, we compared the odds ratio of the associations between resilience and ED to the odds ratio of the associations between depressive symptomatology (using the CES-D [71]) and ED, since depression is a well-known risk factor for ED [7,11]. In our study, the OR for incident ED comparing participants without depressive symptomatology to participants with depressive symptomatology, was 0.41 (95%CI: 0.36, 0.47). Thus, an OR of 0.68 (0.62, 0.74) for incident ED (for a one-point increase in resilience) is probably meaningful at a population level.

## 5. Conclusions

This study explored the cross-sectional and longitudinal associations between resilience and EDs, in a large and heterogeneous sample of French adults. We found that more resilient participants were less likely to have had persistent EDs during the three-year follow-up, and in particular they were less likely to have restrictive, bulimic or hyperphagic disorders. They were also less likely to have incident ED, and in particular bulimic, hyperphagic or other EDs at follow-up. In addition, participants with higher resilience were more likely to recover from EDs over this period than to have a persistent ED, suggesting that the promotion of resilience may help recovery. Our results indicate a potential protective effect of resilience on EDs and could therefore suggest a promising psychological orientation to be integrated into public health programs aiming at preventing EDs. Future longitudinal and interventional studies with different ED measures are needed to confirm these findings, and in particular intervention studies.

## Figures and Tables

**Table 1:** Descriptive characteristics of the participants and the respective resilience (BRS) scores ^1^.

	All (N = 25,000)	Resilience (BRS) ^2^	*p* Value ^3^
Full sample		3.33 ± 0.69 ^4^	
Age (years)	55.01 ± 13.56	0.10 (0.09, 0.11) ^5^	<0.0001
Sex (%)			0.044
Men	25.72	3.49 ± 0.67	
Women	74.28	3.28 ± 0.68	
Educational level (%)			0.0035
Primary	1.94	3.38 ± 0.70	
Secondary	28.09	3.34 ± 0.69	
Undergraduate	31.76	3.31 ± 0.69	
Postgraduate	37.42	3.35 ± 0.67	
Missing data	0.79		
Occupational status (%)			<0.0001
Unemployed	8.14	3.18 ± 0.76	
Student	0.82	3.09 ± 0.71	
Self-employed, farmer	1.71	3.51 ± 0.68	
Employee, manual worker	12.48	3.21 ± 0.71	
Intermediate professions	14.42	3.28 ± 0.67	
Managerial staff, intellectual professions	23.04	3.38 ± 0.67	
Retired	37.59	3.40 ± 0.66	
Missing data	1.80		
Equivalized monthly household income (%)			<0.0001
<1200€	8.58	3.23 ± 0.74	
1200–1799€	18.82	3.31 ± 0.70	
1800–2699€	25.50	3.34 ± 0.68	
≥2700€	33.60	3.41 ± 0.66	
Unwilling to answer	11.29	3.26 ± 0.68	
Missing data	2.21		
Family situation			<0.0001
Living alone without children	10.97	3.18 ± 0.72	
Living alone with children	14.30	3.39 ± 0.71	
Living in a couple without children	11.06	3.27 ± 0.69	
Living in a couple with children	63.24	3.36 ± 0.67	
Missing data	0.43		
Smoking (%)			<0.0001
Current smoker	9.63	3.37 ± 0.69	
Former smoker	39.34	3.37 ± 0.68	
Never smoker	50.62	3.3 ± 0.69	
Missing data	0.41		
Physical activity (%)			<0.0001
Low	21.60	3.24 ± 0.71	
Moderate	39.99	3.3 ± 0.67	
High	38.25	3.42 ± 0.67	
Missing data	0.17		
BMI (2017) (%)			<0.0001
Underweight (<18.5 kg/m^2^)	4.15	3.19 ± 0.69	
Normal weight (18.5–24.9 kg/m^2^)	62.03	3.33 ± 0.67	
Overweight (25–29.9 kg/m^2^)	24.80	3.38 ± 0.69	
Obesity class I (30–34.9 kg/m^2^)	6.54	3.33 ± 0.75	
Obesity class II (35–39.9 kg/m^2^)	1.85	3.25 ± 0.79	
Obesity class III (≥40 kg/m^2^)	0.62	3.18 ± 0.77	
BMI (2020) (%)			<0.0001
Underweight (<18.5 kg/m^2^)	4.42	3.17 ± 0.7	
Normal weight (18.5–24.9 kg/m^2^)	60.37	3.33 ± 0.67	
Overweight (25–29.9 kg/m^2^)	25.11	3.37 ± 0.69	
Obesity class I (30–34.9 kg/m^2^)	7.32	3.30 ± 0.74	
Obesity class II (35–39.9 kg/m^2^)	1.99	3.26 ± 0.80	
Obesity class III (≥40 kg/m^2^)	0.79	3.18 ± 0.80	
Eating disorders (2017) (%) ^6,7^			<0.0001
No	90.95	3.36 ± 0.67	
Yes	9.05	3.06 ± 0.74	
Categories of eating disorders (2017) (%) ^6,7^			<0.0001
No eating disorders	90.95	3.36 ± 0.67	
Restrictive disorders	0.68	2.98 ± 0.71	
Bulimic disorders	2.30	3.02 ± 0.71	
Hyperphagic disorders	4.82	3.06 ± 0.76	
Other type of eating disorders	1.25	3.18 ± 0.73	
Eating disorders (2020) (%) ^6^			<0.0001
No	92.40	3.36 ± 0.68	
Yes	7.60	3.06 ± 0.72	
Categories of eating disorders (2020) (%) ^6,7^			<0.0001
No eating disorders	92.40	3.36 ± 0.68	
Restrictive disorders	0.51	2.98 ± 0.72	
Bulimic disorders	1.94	3.02 ± 0.71	
Hyperphagic disorders	3.79	3.05 ± 0.74	
Other type of eating disorders	1.35	3.15 ± 0.68	
Eating disorders (2017 and 2020) ^6^			<0.0001
No eating disorders (2017: no ED, 2020: no ED)	85.72	3.37 ± 0.67	
Incident (2017: no ED, 2020: ED)	4.08	3.16 ± 0.69	
Persistent (2017: ED, 2020: ED)	4.68	2.94 ± 0.74	
Recovery (2017: ED, 2020: no ED)	5.52	3.14 ± 0.72	
Intermittent (incident or recovery)	9.60	3.13 ± 0.73	

Abbreviations: BRS, Brief Resilience Scale; EDs, eating disorders; SCOFF, Sick-Control-One-Fat-Food Questionnaire. ^1^ NutriNet-Santé study, 2017, N = 25,000. ^2^ Score ranges from 1 to 5. The highest score corresponds to the highest resilience. ^3^ All *p*-Value based on Pearson correlation for continuous variables and Student *t* test, and variance analyses (ANOVA) for categorical variables. ^4^ Mean ± SD, all such values. ^5^ Pearson correlations (95% CI), all such values. ^6^ Eating disorders were assessed with the SCOFF questionnaire. ^7^ The Expali™ algorithm [44] was used to distinguish the different ED categories. It takes into account each SCOFF response and the BMI to split participants into four broad categories based on the Diagnostic and Statistical Manual of Mental Disorders, Fifth Revision (DSM-5) categories of ED.

**Table 2:** Cross-sectional associations between resilience (BRS) (independent variable) and eating disorders (EDs) (SCOFF questionnaire) (dependent variable) in 2017 ^1^.

	Model 1 ^2^		Model 2 ^3^	
	Resilience OR (95% CI)	*p* ^4^	Resilience OR (95% CI)	*p* ^4^
Eating disorders ^5^				
No (N = 22,737)	Ref		Ref	
Yes (N = 2263)	0.53 (0.5, 0.56)	<0.0001	0.58 (0.55, 0.62)	<0.0001
Categories of Eating Disorders ^5,6^				
No eating disorder (N = 22,737)	Ref		Ref	
Restrictive disorders (N = 170)	0.45 (0.36, 0.55)	<0.0001	0.53 (0.43, 0.66)	<0.0001
Bulimic disorders (N = 575)	0.49 (0.43, 0.55)	<0.0001	0.56 (0.49, 0.63)	<0.0001
Hyperphagic disorders (N = 1206)	0.52 (0.48, 0.57)	<0.0001	0.57 (0.52, 0.62)	<0.0001
Other eating disorders (N = 312)	0.68 (0.58, 0.8)	<0.0001	0.73 (0.62, 0.86)	0.0002

Abbreviations: BRS, Brief resilience Scale; SCOFF, Sick-Control-One-Fat-Food Questionnaire. ^1^ NutriNet-Santé study, 2017, N= 25,000. ^2^ Model 1: Unadjusted. ^3^ Model 2: Adjusted for age, gender, educational level, occupational status, equivalized monthly household income, family situation, smoking status and physical activity. ^4^ *p* value based on binary (yes vs. no) or multinomial (categories of EDs) logistic regression with resilience as a continuous independent variable and EDs as categorical dependent variables. ^5^ Eating disorders were assessed with the SCOFF questionnaire. ^6^ The Expali™ algorithm [44] was used to distinguish the different ED categories. It takes into account each SCOFF response and the BMI to split participants into four broad categories based on the Diagnostic and Statistical Manual of Mental Disorders, Fifth Revision (DSM-5) categories of ED.

**Table 3:** Longitudinal associations between resilience (BRS) (independent variable) and eating disorders (EDs) (SCOFF questionnaire) (dependent variable) ^1^.

	Model 1 ^2^		Model 2 ^3^	
	Resilience OR (95% CI)	*p* ^4^	Resilience OR (95% CI)	*p* ^4^
Eating disorders ^5^				
No eating disorder (N = 21,703)	Ref		Ref	
Incident (N = 1034)	0.63 (0.57, 0.69)	<0.0001	0.68 (0.62, 0.74)	<0.0001
Persistent (N = 866)	0.40 (0.36, 0.44)	<0.0001	0.46 (0.42, 0.51)	<0.0001
Intermittent (N = 2431)	0.61 (0.58, 0.65)	<0.0001	0.66 (0.62, 0.71)	<0.0001
Category of eating disorder ^5,6^				
No eating disorders (N = 21,703)	Ref		Ref	
Restrictive disorders				
Incident ^7^ (N = 50)	0.58 (0.39, 0.87)	0.0091	0.72 (0.47, 1.08)	0.11
Persistent ^8^ (N = 78)	0.36 (0.26, 0.49)	<0.0001	0.44 (0.32, 0.61)	<0.0001
Intermittent ^9^ (N = 142)	0.54 (0.43, 0.68)	<0.0001	0.63 (0.50, 0.81)	0.0002
Bulimic disorders				
Incident ^7^ (N = 230)	0.64 (0.53, 0.77)	<0.0001	0.71 (0.58, 0.86)	0.0004
Persistent ^8^ (N = 256)	0.36 (0.30, 0.43)	<0.0001	0.41 (0.34, 0.49)	<0.0001
Intermittent ^9^ (N = 549)	0.62 (0.55, 0.70)	<0.0001	0.69 (0.61, 0.78)	<0.0001
Hyperphagic disorders				
Incident ^7^ (N = 480)	0.63 (0.55, 0.72)	<0.0001	0.68 (0.59, 0.77)	<0.0001
Persistent ^8^ (N = 468)	0.41 (0.36, 0.46)	<0.0001	0.46 (0.40, 0.52)	<0.0001
Intermittent ^9^ (N = 1218)	0.61 (0.56, 0.66)	<0.0001	0.65 (0.59, 0.71)	<0.0001
Other eating disorders				
Incident ^7^ (N = 274)	0.61 (0.51, 0.73)	<0.0001	0.65 (0.54, 0.77)	<0.0001
Persistent ^8^ (N = 64)	0.67 (0.47, 0.96)	0.03	0.75 (0.52, 1.07)	0.11
Intermittent ^9^ (N = 522)	0.64 (0.56, 0.72)	<0.0001	0.67 (0.59, 0.76)	<0.0001

Abbreviations: BRS, Brief resilience Scale; SCOFF, Sick-Control-One-Fat-Food Questionnaire. ^1^ NutriNet-Santé study, 2017–2020, N = 25,000. ^2^ Model 1: Unadjusted. ^3^ Model 2: Adjusted for age, gender, educational level, occupational status, equivalized monthly household income, family situation, smoking status and physical activity. ^4^ *p* value based on binary (yes vs. no) or multinomial (categories of EDs) logistic regression with resilience as a continuous independent variable and EDs as categorical dependent variables. ^5^ Eating disorders were assessed with the SCOFF questionnaire. ^6^ The Expali™ algorithm was used to distinguish the different ED categories. It takes into account each SCOFF response and the BMI to split participants into four broad categories based on the Diagnostic and Statistical Manual of Mental Disorders, Fifth Revision (DSM-5) categories of ED. ^7^ Incident: having no ED in 2017 but an ED in 2020. ^8^ Persistent: having the same ED in 2017 and 2020. ^9^ Intermittent: having an ED either in 2017 or 2020.

**Table 4:** Longitudinal associations between resilience (BRS) (independent variable) and eating disorders (EDs) (SCOFF) (dependent variable) in 2263 participants ^1^.

	Model 1 ^2^		Model 2 ^3^	
	Resilience OR (95% CI)	*p* ^4^	Resilience OR (95% CI)	*p* ^4^
Eating disorders ^5^				
Recovered from eating disorder ^6^ (N = 1397)	Ref		Ref	
Persistent eating disorder ^7^ (N = 866)	0.70 (0.62, 0.78)	<0.0001	0.73 (0.65, 0.82)	<0.0001
Category of eating disorders ^5,8^				
Recovered from eating disorders ^9,6^ (N = 1397)	Ref		Ref	
Persistent restrictive disorders ^7^ (N = 78)	0.63 (0.46, 0.85)	0.0029	0.70 (0.50, 0.97)	0.033
Persistent bulimic disorders ^7^ (N = 256)	0.63 (0.52, 0.75)	<0.0001	0.65 (0.54, 0.79)	<0.0001
Persistent hyperphagic disorders ^7^ (N = 468)	0.70 (0.61, 0.81)	<0.0001	0.73 (0.63, 0.84)	<0.0001
Persistent other eating disorders ^7^ (N = 64)	1.10 (0.78, 1.56)	0.57	1.11 (0.78, 1.57)	0.57

Abbreviations: BRS, Brief resilience Scale; SCOFF, Sick-Control-One-Fat-Food Questionnaire. ^1^ NutriNet-Santé study, 2017–2020, N = 2263. ^2^ Model 1: Unadjusted. ^3^ Model 2: Adjusted for age, gender, educational level, occupational status, equivalized monthly household income, family situation, smoking status and physical activity. ^4^ *p* value based on binary (yes vs. no) or multinomial (categories of EDs) logistic regression with resilience as a continuous independent variable and EDs as categorical dependent variables. ^5^ Eating disorders were assessed with the SCOFF questionnaire. ^6^ Recovery: having an ED in 2017 but no ED in 2020. ^7^ Persistent: having the same ED in 2017 and 2020. ^8^ The Expali algorithm was used to distinguish the different ED categories. It takes into account each SCOFF response and the BMI to split participants into four broad categories based on the Diagnostic and Statistical Manual of Mental Disorders, Fifth Revision (DSM-5) categories of ED. ^9^ Any eating disorders.

## Data Availability

Data described in the manuscript, code book, and analytic code will be made available upon request pending application and approval.
